# Plasma Neurofilament Heavy Chain Levels Correlate to Markers of Late Stage Disease Progression and Treatment Response in SOD1^G93A^ Mice that Model ALS

**DOI:** 10.1371/journal.pone.0040998

**Published:** 2012-07-16

**Authors:** Ching-Hua Lu, Axel Petzold, Bernadett Kalmar, James Dick, Andrea Malaspina, Linda Greensmith

**Affiliations:** 1 Sobell Department of Motor Neuroscience and Movement Disorders, MRC Centre for Neuromuscular Disorders, UCL Institute of Neurology, University College London, London, United Kingdom; 2 Department of Neuroinflammation, UCL Institute of Neurology, University College London, London, United Kingdom; 3 VU Medical Centre, Dept. of Neurology, Amsterdam, The Netherlands; 4 Trauma and Neuroscience Centre, Blizard Institute, Barts and The School of Medicine and Dentistry, Queen Mary University of London, London, United Kingdom; 5 North-East London and Essex MND Care and Research Centre, London, United Kingdom; Baylor College of Medicine, Jiao Tong University School of Medicine, United States of America

## Abstract

**Background:**

Amyotrophic lateral sclerosis (ALS) is an incurable neurodegenerative disorder characterised by progressive degeneration of motor neurons leading to death, typically within 3–5 years of symptom onset. The diagnosis of ALS is largely reliant on clinical assessment and electrophysiological findings. Neither specific investigative tools nor reliable biomarkers are currently available to enable an early diagnosis or monitoring of disease progression, hindering the design of treatment trials.

**Methodology/Principal Findings:**

In this study, using the well-established SOD1^G93A^ mouse model of ALS and a new in-house ELISA method, we have validated that plasma neurofilament heavy chain protein (NfH) levels correlate with both functional markers of late stage disease progression and treatment response. We detected a significant increase in plasma levels of phosphorylated NfH during disease progression in SOD1^G93A^ mice from 105 days onwards. Moreover, increased plasma NfH levels correlated with the decline in muscle force, motor unit survival and, more significantly, with the loss of spinal motor neurons in SOD1 mice during this critical period of decline. Importantly, mice treated with the disease modifying compound arimoclomol had lower plasma NfH levels, suggesting plasma NfH levels could be validated as an outcome measure for treatment trials.

**Conclusions/Significance:**

These results show that plasma NfH levels closely reflect later stages of disease progression and therapeutic response in the SOD1^G93A^ mouse model of ALS and may potentially be a valuable biomarker of later disease progression in ALS.

## Introduction

Amyotrophic lateral sclerosis (ALS) is a fatal neurodegenerative disorder characterised by progressive degeneration of motor neurons in the motor cortex, brain stem and spinal cord, leading to paralysis and death, typically within 3–5 years from symptom onset. Riluzole is the only FDA-approved treatment for ALS, which prolongs median survival by only 2–3 months in patients treated for at least 18 months [1]. Importantly, the greatest benefit of Riluzole is observed when treatment is initiated early in the course of the disease, highlighting the importance of early intervention in ALS [2]. In the absence of a reliable diagnostic biomarker, the recognition of ALS relies largely on clinical assessment and electrophysiological findings, which provide evidence of upper and lower motor neuron involvement [3]. The lack of more specific investigative tools and of easily measurable biomarkers typically results in a 12–14 month delay between symptom onset to diagnosis, for both sporadic (sALS) and familial ALS (fALS) [4]. This delay not only prevents patients from receiving early administration of the only available therapy, Riluzole, but also impedes their early recruitment to clinical trials, thereby reducing the likelihood of success of potential disease-modifying agents. Therefore, there is an urgent need to develop biomarkers for ALS, to both speed up diagnosis and to monitor disease progression. This is particularly true for clinical trials, where such a biomarker would be invaluable for statistical power and as an indicator of both positive and negative responses to treatment.

Although the precise underlying pathology of ALS is not yet fully understood, a number of molecular mechanisms have been identified that are present in both human ALS and in mouse models of ALS. Transgenic mice carrying the mutant human *Cu/Zn superoxide dismutase 1(SOD1)* gene, causative for approximately 10–20% of familial ALS cases [5], have a disease phenotype that resembles that of ALS patients including progressive motor neuron degeneration accompanied by gradual muscle paralysis and premature death [6]. These mice have been invaluable in identifying several pathological mechanisms that contribute to ALS. ALS is now known to be a multifactorial disorder that involves excitotoxicity, dysfunctional RNA metabolism, mitochondrial dysfunction, endoplasmic reticulum stress, proteasomal dysfunction, activation of inflammatory pathways and, importantly for this study, impaired axonal transport and protein aggregation [7]. Several proteins have been shown to aggregate in tissue of ALS patients and animal models of ALS including structural proteins of the axonal cytoskeleton such as neurofilaments [8]. Despite the complexity of ALS pathogenesis, the multitude of molecular mechanisms involved in the disease process not only provide targets for development of novel therapeutics but may also provide clues for the development of biomarkers of disease progression.

The neurofilament proteins (Nfs) are a major component of the cytoskeleton and play an important role in the maintenance of axonal calibre [9]. Nfs are assembled into a unique heteropolymer structure with a specific stoichiometric composition of at least four subunits: neurofilament light chain (NfL, 68 kDa), neurofilament medium chain (NfM, 150 kDa), neurofilament heavy chain (NfH, 200 kDa) and alpha-internexin (INA, 66 kDa) [10]. The three neurofilament subunits differ mainly in the length of the C-terminal tail domain, which results in different states of phosphorylation and susceptibility to proteases [11]. Mutations in Nfs have been reported in several neurodegenerative diseases, including Alzheimer’s disease (AD), Parkinson’s disease (PD), Charcot-Marie-Tooth Disease, giant axonal neuropathy, diabetic neuropathy, progressive supranuclear palsy, spinal muscular atrophy, as well as ALS [9]. Although only a small number of variants in the NfH gene have been identified in approximately 1% sALS patients [12], [13], one common pathological finding of both sALS and fALS is the accumulation of phosphorylated Nfs in the perikaryon and in axonal spheroids, which are normally only present in distal axons and nerve terminals [14]. Manipulations of the stoichiometry of the three Nf subunits has been shown to facilitate the development of early-onset motor neuron death in transgenic mouse models of ALS [15], [16], [17], thus supporting the critical involvement of Nfs in ALS pathology.

Monitoring of tissue specific components released into biological fluids during disease progression can be used to aid diagnosis and reflect pathological severity [4], [18], [19]. Nfs levels in the cerebrospinal fluid (CSF), being the closest body fluid compartment to the CNS, have been investigated as potential disease biomarkers in several neurological disorders. Recent studies have shown that CSF Nf levels might serve as a prognostic marker in multiple sclerosis [20], may assist in the differential diagnosis between frontotemporal dementia (FTD) and early onset AD [21], and may help distinguish Parkinsonian syndromes in combination with CSF tau levels [22], [23]. Furthermore, CSF Nf levels are much higher in ALS than in other neurodegenerative disorders such as AD [23]–[26] and correlate inversely with disease duration [27]. It has also been suggested that the phosphorylation state of Nfs can be indicative of neuronal pathology and high levels of phosphorylated NfH have been detected in neurodegenerative disorders [9]. However, because of its invasive nature, serial CSF sampling is not tolerated by all patients and CSF is clearly not the ideal biofluid for repeated sampling for the purpose of monitoring progression in ALS. Thus, a functionally validated Nf-based blood biomarker for the longitudinal monitoring of disease development and informative of treatment response would be highly desirable.

Previous studies have identified a crucial analytical problem in accurate, reproducible quantification of NfH levels in blood samples due to the presence of Nf aggregates [28]. These aggregates are a source of endogenous binding for NfH in plasma that leads to a ‘hook effect’ during serial dilutions. To overcome this problem, we recently developed a method in which Nf aggregates are gently disaggregated, allowing accurate and highly sensitive quantification of plasma NfH levels using ELISA [28]. In this study, we have used this method to determine the longitudinal changes in plasma NfH levels in transgenic mice carrying mutant human *SOD1^G93A^* gene during disease progression. Importantly, we have correlated these changes with the decline in neuromuscular function and motor neuron survival. In addition, we undertook a differential analysis of the NfH phosphorylation status. Thus, in separate assays, we examined hyperphosphorylated and variably-phosphorylated NfH levels. Finally, in order to establish the potential of this approach as an outcome measure in clinical trials, we examined whether plasma NfH levels are improved following treatment with arimoclomol, which we have previously shown to modify disease progression in SOD1^G93A^ mice [29], [30], and which is currently in a PhaseII/III clinical trial in mutant SOD1 related fALS patients [31].

## Results

### Plasma NfH Levels in SOD1 Mice Increase Significantly from a Late Symptomatic Stage

Hyperphosphorylated (NfH^SMI34^) and variably-phosphorylated (NfH^SMI35^) NfH levels in plasma from mice in each experimental group were determined at various stages of disease using a 4-layer sandwich ELISA.

**Figure 1:**
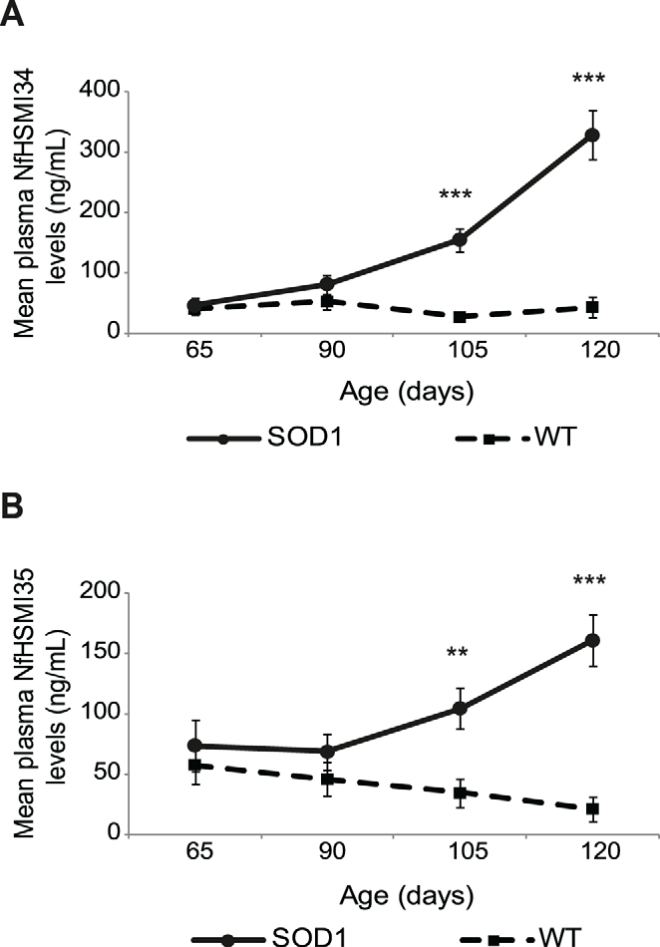
Longitudinal assessment of plasma NfH levels in WT and SOD1 mice. The graphs show the mean plasma level (ng/ml) of **A**) NfH^SMI34^ and **B**) NfH^SMI35^ in SOD1 (circles; n = 19) and WT (squares; n = 13) mice at 65 (pre-symptomatic), 90 (early symptomatic), 105 (late symptomatic), and 120 (end stage) days of age. Error bars  =  S.E.M. Friedman test was used for analysis of the pattern of plasma NfH levels during disease progression, and Mann-Whitney Test was then used for group comparison at each time point. **p = 0.002, ***p<0.0001.

As can be seen in Fig. 1A, in WT mice, the mean plasma levels of hyperphosphorylated NfH^SMI34^ remained unchanged during the duration of the study, up to 120 days of age (Friedman test, p = 0.518). In contrast, in SOD1 mice, there was a significant change in NfH^SMI34^ levels during overall disease progression (Friedman test, p<0.0001). Plasma NfH^SMI34^ levels were higher in SOD1 mice than in WT littermates at all ages examined, although this difference only reached statistical significance from 105 days of age onwards (65 days: SOD1 46.2±13.1, WT 40.6±8.9; 90 days: SOD1 81.8±15.8, WT 52.7±12.2; 105 days: SOD1 155.4±19.3, WT 26.7±6.8, p<0.0001; 120 days: SOD1 328.4±40.3, WT 43.5±17.3, p<0.0001; mean ± S.E.M ng/mL; Mann-Whitney test; Fig. 1A).

The mean levels of plasma NfH^SMI35^ (Fig. 1B), the variably-phosphorylated NfH which is considered to be the less pathological form of NfH, also remained stable in WT mice throughout the study period (Friedman test, p = 0.405). In contrast, in SOD1 mice, plasma NfH^SMI35^ levels increased significantly throughout disease progression (Friedman test, p<0.0001), although not as dramatically as NfH^SMI34^, the more pathological phosphoform, and became significantly higher than WT from 105 days of age onwards (65 days: SOD1 74.0±20.8, WT 58.0±16.0; 90 days: SOD1 68.8±14.8, WT 46.2±14.2; 105 days, SOD1 104.8±17.0, WT 34.7±11.6, p = 0.002; 120 days, SOD1 161.0±21.5, WT 21.6±9.9, p<0.0001; mean ± S.E.M ng/mL; Mann-Whitney test; Fig. 1B).

In addition, in WT mice, a comparison of NfH^SMI35^ and NfH^SMI34^ levels showed that plasma levels of NfH were not only low, but that there was also no difference in the relative levels of the two NfH phosphoforms at any age examined (Mann-Whitney test, p = 0.479, 0.353, 0.817, 0.565 at 65, 90, 105, and 120 days, respectively; data not shown). In contrast, in SOD1 mice, a comparison of the relative levels of the two NfH phosphoforms revealed that levels of NfH^SMI34^ compared with NfH^SMI35^ increased as disease advanced (comparison NfH^SMI34^: NfH^SMI35^ at 65 days: p = 0.507; 90 days: p = 0.546; 105 days: p = 0.068; 120 days: p = 0.004; Mann-Whitney Test). Thus, in SOD1 mice plasma levels of the more pathological NfH^SMI34^ phosphoform increased to a greater extent than the less pathological NfH^SMI35^.

### Correlations between Plasma NfH Levels and Functional Measures of Disease Progression in SOD1 Mice

#### Increased plasma NfH levels inversely correlate with grip strength in SOD1 mice

**Figure 2:**
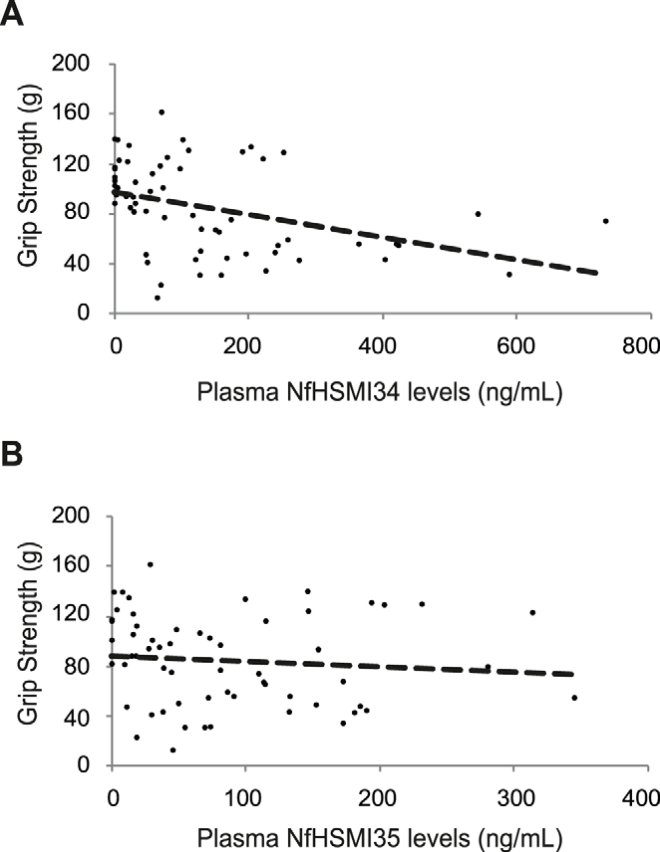
The correlation between grip strength and plasma NfH levels in SOD1 mice during disease progression. The graphs show the relationship between mean grip strength (g) and plasma NfH phosphoform levels (ng/mL) in individual SOD1 mice (n = 16) at 65, 90, 105, and 120 days of age. **A**) There is a strong inverse correlation between plasma NfH^SMI34^ levels and grip strength in SOD1 mice (Spearman’s rho = −0.583, p<0.0001). The best-fit line (dashed line) is shown. **B**) There is a moderate inverse correlation between plasma NfH^SMI35^ and grip strength in SOD1 mice (Spearman’s rho = −0.335, p<0.0001). The best-fit line (dashed line) is shown.

In order to establish the relationship between the changes in plasma NfH levels detected in SOD1 mice and the decline in neuromuscular function that occurs as disease progresses in these mice, a number of functional outcome measures were investigated. Firstly, at each time point, grip strength and body weight was determined in each mouse prior to blood collection. The analysis of the correlation between plasma NfH levels and the corresponding mean grip strength is summarised in Fig. 2. The results show that in SOD1 mice, there was a moderate-to-strong inverse correlation between plasma NfH levels and grip strength at all time points examined, 65, 90, 105 and 120 days (Spearman’s rho (R) : NfH^SMI34^ v.s. GS: −0.583, p<0.0001; NfH^SMI35^ v.s. GS: −0.335, p<0.0001; Fig. 2A & 2B). No such correlation in plasma NfH levels and grip strength was observed in WT mice at any age examined (data not shown).

#### Increased plasma NfH levels correlate with the decline in isometric muscle force in hindlimb muscles of SOD1 mice

In order to obtain a more detailed assessment of the relationship between plasma NfH levels and quantitative functional markers of disease progression, an *in vivo* physiological assessment of isometric muscle force in the hindlimb muscles of SOD1 mice was undertaken. This physiological assessment of isometric muscle force of specific muscles provides a more sensitive and quantitative assessment of functional decline than grip strength analysis, which can only detect general motor deficits in SOD1 mice relatively late in the disease process, when many motor neurons have already died. We have previously generated similar physiological data in our lab in SOD1 mice at various stages of disease ranging from early symptomatic (90 days) to end-stage disease (130 days) [29], [30], [32]. We then examined whether plasma NfH levels correlate with this profile of functional decline in SOD1 mice.

**Figure 3:**
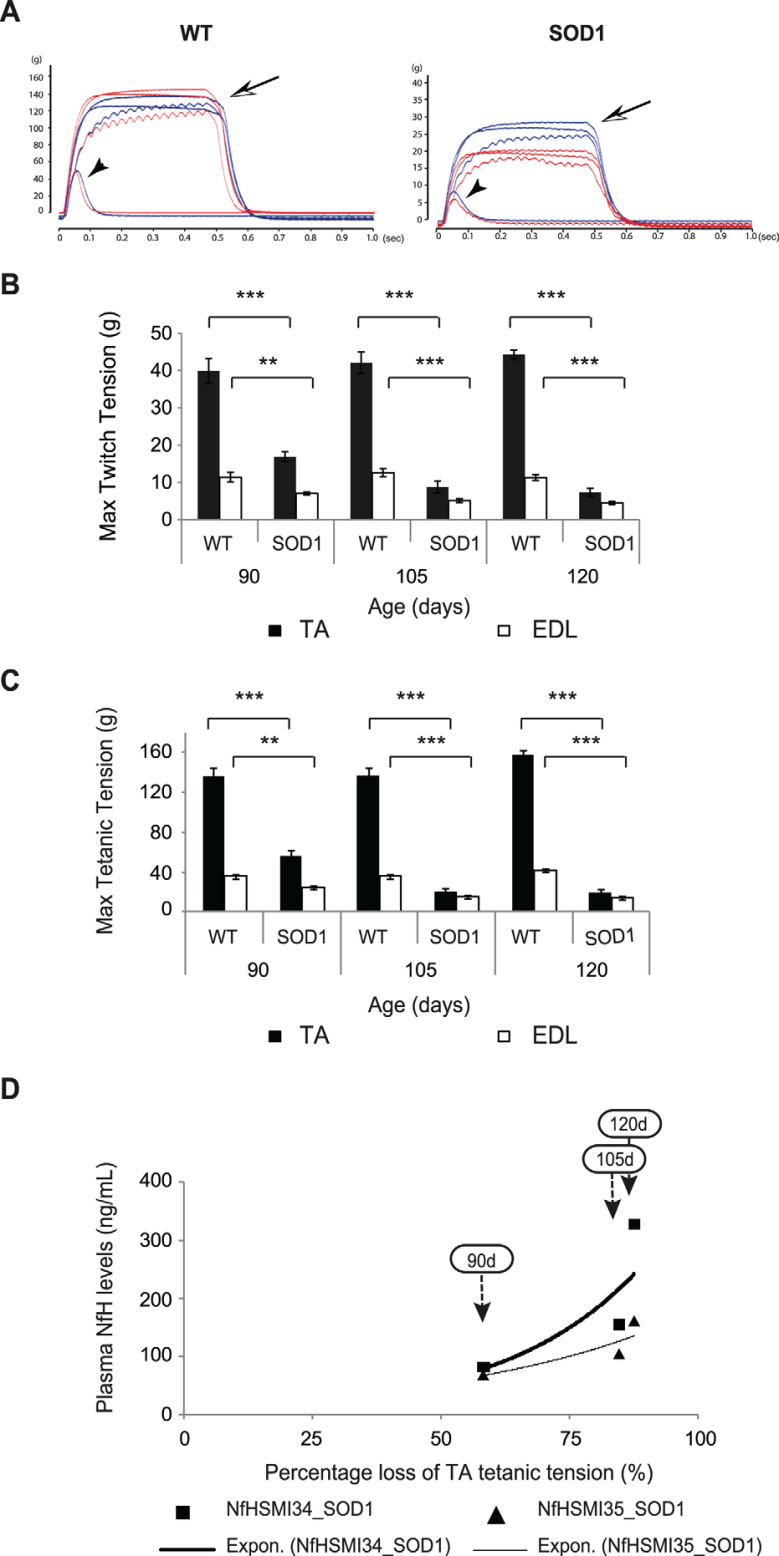
The correlation between plasma NfH phosphoform levels and hindlimb muscle force in SOD1 mice during disease progression. (**A**) Examples of recordings of maximum tetanic (arrow) and twitch (arrow head) tension in TA muscles of WT and vehicle treated SOD1 mice at 105 days of age. (**B**) The bar chart shows the mean maximum twitch tension (g) of TA (filled bars) and EDL (open bars) muscles. (**C**) The bar chart shows the mean maximum tetanic tension (g) of TA (filled bars) and EDL (open bars) muscles. (**D**)The graph shows the percentage loss of TA maximum tetanic tension, relative to WT, and the corresponding plasma NfH^SMI34^ (black squares) and NfH^SMI35^ (black triangles) levels in vehicle treated SOD1 mice at each stage of disease (indicated by arrows). Exponential regression lines are shown for NfH^SMI34^ (thick black line) and NfH^SMI35^ (thin black line). Error bars  =  S.E.M. Mann-Whitney Test: *p<0.05; **p<0.01; ***p<0.0001.

Typical examples of tetanic and twitch force traces from the TA muscles of 105-day-old WT and SOD1 mice are shown in Fig. 3A. The mean maximum twitch and tetanic force of the Tibialis Anterior (TA) and Extensor Digitorum Longus (EDL) muscles of WT and SOD1 at various stages of disease is summarised in Fig. 3B and 3C, respectively, which shows the well established, progressive decrease in muscle force in both TA and EDL muscles that occurs in SOD1 mice as they age. The results also show that in SOD1 mice, TA muscles were affected earlier in disease and to a greater extent than EDL muscles. Thus, the maximum tetanic force (Fig. 3C; mean±S.E.M.) in SOD1 TA was 56.7±5 g (n = 13) at 90 days, 21.1±3 g (n = 16) at 105 days and 19.5±3.5 g (n = 12) at 120 days. When expressed as a percentage of WT TA muscle force in age-matched WT mice, the force produced by SOD1 TA was 41.7% at 90 days, 15.3% at 105 days and 12% at 120 days (p<0.0001 at each time point; Mann-Whitney test). The decline in force in SOD1 EDL muscles occurred at a steadier, slower pace. Thus, in SOD1 EDL muscles the force produced was 24.7±1.5 g (n = 15) at 90 days, 15.7±1.6 g (n = 19) at 105 days and 13.7±1.0 g (n = 11) at 120 days, which was 68.6%, 43.9% and 33.3% of the force produced in WT EDL muscles at the respective ages (p<0.01; <0.0001; <0.0001, respectively; Mann-Whitney test). A similar pattern of decline was also found in the twitch tension of TA and EDL muscles of SOD1 mice (Fig. 3B).

As shown in Fig. 3D, an exponential regression analysis of the decline in maximum TA muscle force at 90, 105 and 120 days of age (indicated by the respective arrows) and their corresponding plasma NfH^SMI34^ (black squares) or NfH^SMI35^ (black triangles) levels in SOD1 mice was undertaken. It can be seen that there is a moderate-strong correlation between muscle force and plasma NfH levels in SOD1 mice (R^2^ = 0.79 and 0.82 for NfH^SMI34^ and NfH^SMI35^ respectively).

#### Increased plasma NfH levels correlate with motor unit loss in SOD1 mice

**Figure 4:**
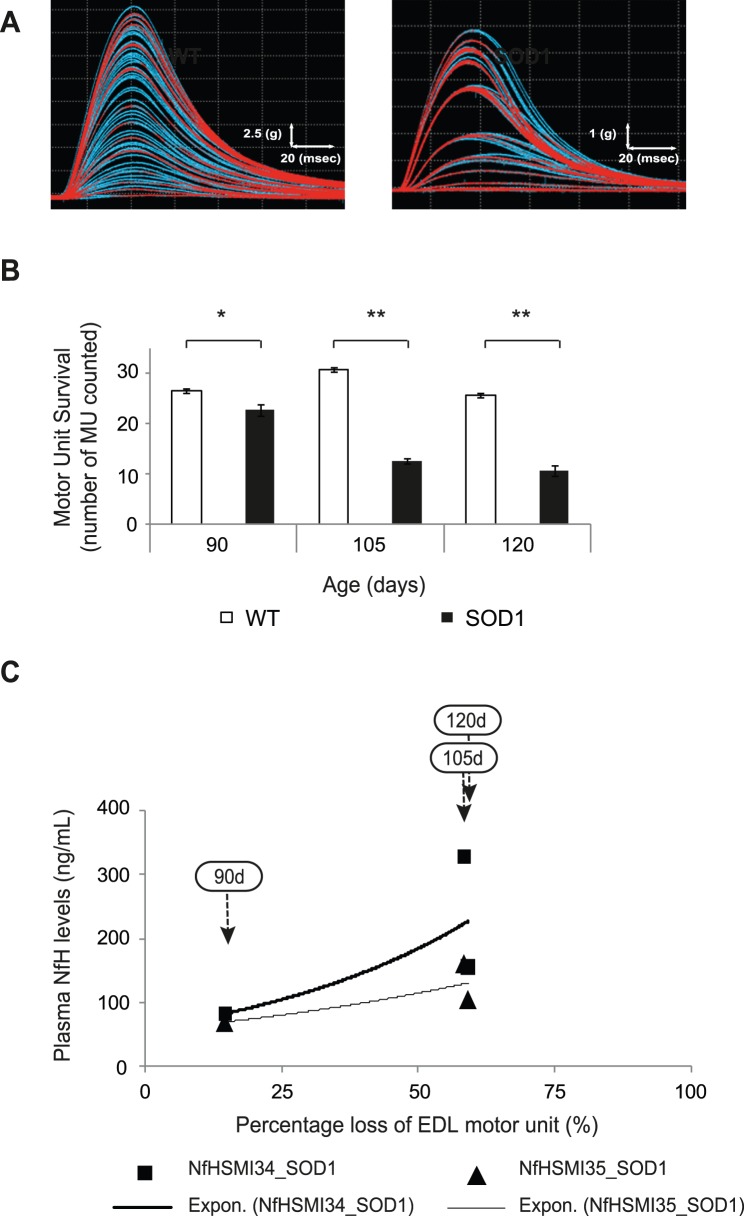
The correlation between plasma NfH phosphoform levels and motor unit survival in EDL muscles in SOD1 mice during disease progression. (**A**) Typical examples of motor unit traces in the EDL muscle of a WT and a vehicle treated SOD1 mouse at 105 days of age. (**B**) The bar chart shows the mean motor unit survival (numbers) in EDL muscles of WT (open bars) and vehicle treated SOD1 (filled bars) mice. (**C**) The percentage loss of EDL motor units, relative to WT, and plasma NfH^SMI34^ (black squares) and NfH^SMI35^ (black triangles) levels in SOD1 mice at each stage of disease (indicated by arrows). Exponential regression lines are shown for NfH^SMI34^ (thick black line) and NfH^SMI35^ (thin black line). Error bars  =  S.E.M. Mann-Whitney Test: *p<0.05; **p<0.01; ***p<0.0001.

Examples of typical motor unit traces from the EDL muscle of 105-day-old SOD1 and WT mice are shown in Fig. 4A. The number of functional motor units that survived in EDL muscles was also determined in SOD1 mice at 90, 105 and 120 days of age and the results are summarised in Fig. 4B. In WT mice, the number of motor units innervating EDL muscles remained constant over the course of the study and the number of motor units innervating EDL (mean±S.E.M.) was found to be 26.6±0.4 (n = 14) at 90 days, 30.8±0.4 (n = 10) at 105 days and 25.7±0.5 (n = 6) at 120 days. In contrast, in SOD1 mice, a significant number of motor units had already died by 90 days, with 22.7±1.2 (n = 17) motor units innervating EDL, a 15% decline compared with WT (p<0.05). Between 90–105 days, there was a 60% decrease in the number of motor units innervating EDL muscles of SOD1 mice and only 12.6±0.5 (n = 15) motor units innervated EDL (p<0.01). This decline in motor unit survival in SOD1 mice plateaued at this level, so that by 120 days, 10.7±1.0 (n = 11; p<0.01) motor units innervated EDL.

Fig. 4C shows the results of an exponential regression analysis of the decline in motor unit survival in EDL muscles at 90, 105 and 120 days (indicated by the respective arrows) and the corresponding plasma NfH^SMI34^ (black squares) or NfH^SMI35^ (black triangles) levels in SOD1 mice. Motor unit loss, expressed as a percentage of motor unit survival in age matched WT mice, can be fitted into an exponential regression with R^2^ = 0.70 for NfH^SMI34^ and R^2^ = 0.73 for NfH^SMI35^, indicating a modest correlation between the extent of EDL motor unit loss and plasma NfH levels in SOD1 mice.

#### Increased plasma NfH levels directly correlate with the extent of motor neuron death in the spinal cord of SOD1 mice

**Figure 5:**
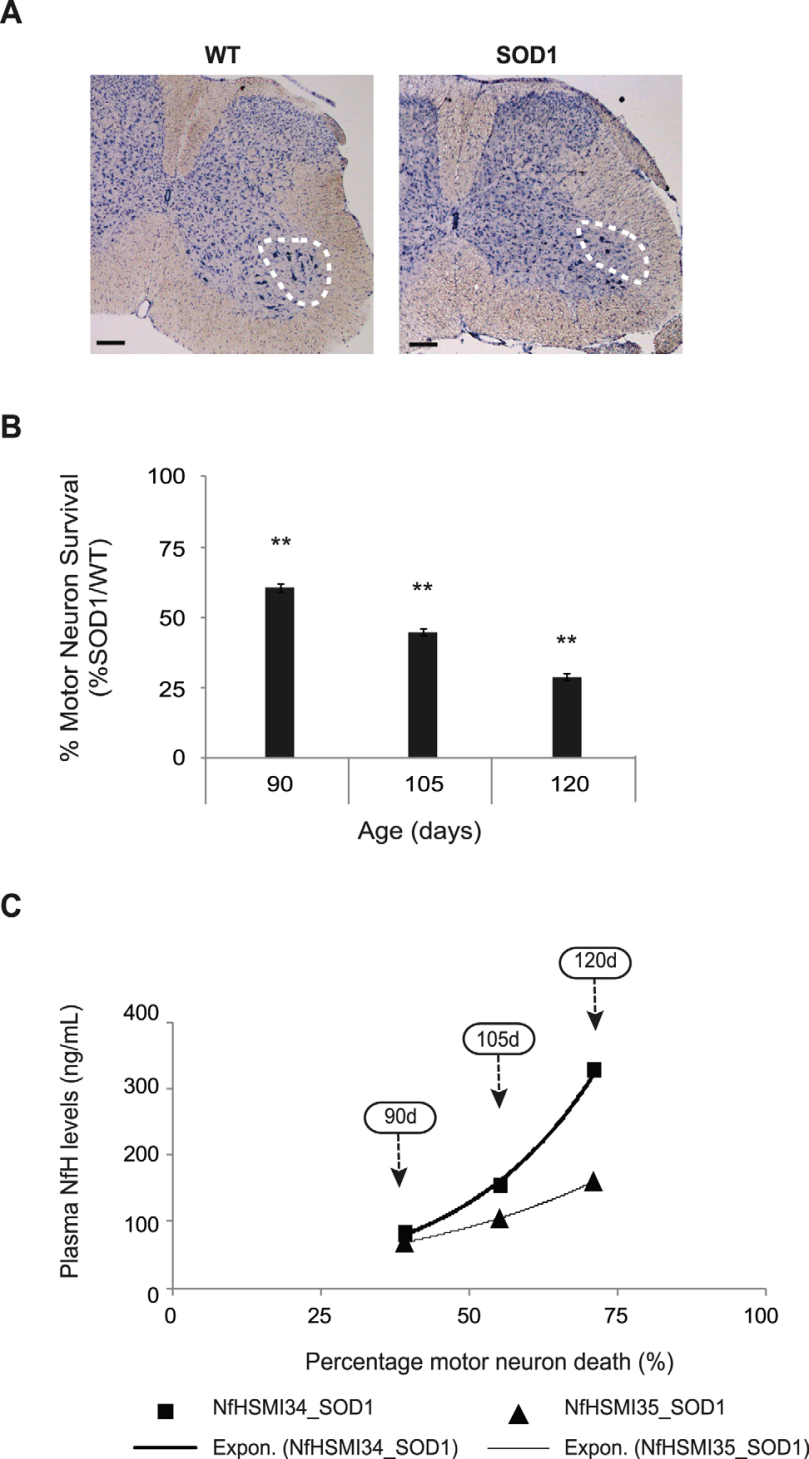
The correlation between plasma NfH phosphoform levels and motor neuron survival in SOD1 mice during disease progression. (**A**) The photomicrographs show cross-sections of the lumbar region at L5 level of spinal cords from a WT and a vehicle treated SOD1 mouse at 105 days of age stained for Nissl. The location of the sciatic motor pool is indicated within the broken circle. Magnificent: 5X. Scale bar = 250 µm. (**B**) The bar chart shows the mean motor neuron survival in SOD1 mice expressed as a percentage of that in age-matched WT littermates at various stages of disease. (**C**) The percentage motor neuron death in SOD1 mice and levels of plasma NfH^SMI34^ (black squares) and NfH^SMI35^ (black triangles) at each disease stage (indicated by arrows). Exponential regression lines are shown for NfH^SMI34^ (thick black line) and NfH^SMI35^ (thin black line). Error bars = S.E.M. Mann-Whitney Test: *p<0.05; **p<0.01; ***p<0.0001.

The defining disease characteristic in both ALS patients and in mouse models is motor neuron degeneration. Therefore, in order to determine if changes in plasma NfH levels in SOD1 mice were a good reflection of the extent of motor neuron degeneration, we next established the extent of motor neuron survival in the lumbar spinal cord of WT and SOD1 mice at various stages of disease. Examples of Nissl stained spinal cord sections, showing the lumbar ventral horn of 105 day old WT and SOD1 mice are shown in Fig. 5A. The results are summarised in Fig. 5B which shows the mean motor neuron survival in 90, 105 and 120 day old SOD1 mice expressed as a percentage of WT. It can be seen that by 90 days, a significant number of motor neurons have already died in SOD1 mice and only 60.8±1.4% of motor neurons survive compared with WT littermates (p<0.01). Motor neurons continue to die in SOD1 mice as disease progresses, so that by 105 days, 44.8±1.2% of motor neurons survive (p<0.01) and by 120 days this is further reduced and only 29.0±1.3% of motor neurons survive (p<0.01; Mann-Whitney test).

In order to establish whether the changes detected in plasma NfH levels correlate with the extent of motor neuron degeneration in SOD1 mice, an exponential regression analysis of motor neuron survival and plasma NfH levels was undertaken. Figure 5C summarises the results and shows the decline in motor neuron survival in SOD1 mice at 90, 105 and 120 days of age (indicated by the respective arrows) and corresponding plasma NfH^SMI34^ (black squares) or NfH^SMI35^ (black triangles) levels. It can be seen that the decline in motor neuron survival observed in SOD1 mice during disease progression fits into an exponential regression with R^2^ = 0.99 for both NfH^SMI34^ and NfH^SMI35^ (p-value of coefficient: 0.03 and 0.006, respectively). This analysis shows that there is a very strong correlation between the extent of motor neuron death and plasma levels of both NfH^SMI34^ and NfH^SMI35^ in SOD1 mice.

### Plasma NfH Levels Reflect the Disease-modifying Effects of Arimoclomol in SOD1 Mice

Since plasma NfH levels appear to be a good reflection of the decline in neuromuscular function in SOD1 mice, we next examined whether plasma NfH levels could reflect the improvements observed in SOD1 mice following treatment with a disease modifying therapy. We therefore examined the effect of treatment of SOD1 mice with arimoclomol, which we have previously shown to significantly delay disease progression and extend the lifespan of SOD1 mice [29], [30]. Therefore, in this study a separate group of SOD1 mice were treated with arimoclomol (10 mg/Kg; i.p; SOD1+A) from 35 days and their plasma was collected at 65, 90, 105 and 120 days of age for analysis of NfH levels.

**Figure 6:**
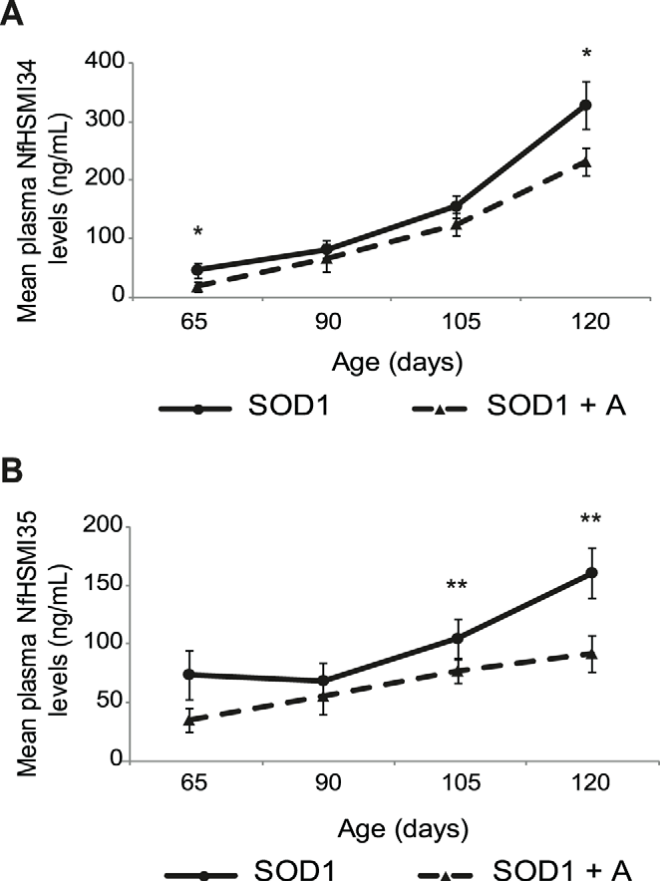
Longitudinal assessment of plasma NfH levels in arimoclomol treated SOD1 mice. The graphs show the mean plasma level (ng/ml) of **A**) NfH^SMI34^ and **B**) NfH^SMI35^ in vehicle treated SOD1 mice (circles; n = 19) and arimoclomol treated SOD1+A mice (triangles; n = 19), at 65 (pre-symptomatic), 90 (early symptomatic), 105 (late symptomatic), and 120 (end stage) days of age. Error bars  =  S.E.M. A *t*-test was performed for direct comparison of the levels of NfH^SMI34^ in the two groups at 105 and 120 days, and a Mann-Whitney Test was performed for the other time points as the data was not normally distributed. Non-parametric analyses take the median value into account more than the mean value in the statistical analysis. *p<0.05; **p<0.01.

As can be seen in Fig. 6A, even at 65 days, a pre-symptomatic age, the effects of arimoclomol were reflected in a reduction in the plasma mean levels of NfH^SMI34^ in treated SOD1+A mice compared with vehicle treated SOD1 littermates. Thus, at 65 days, the mean ± S.E.M of plasma NfH^SMI34^ levels were 17.8±8.8 ng/mL in SOD1+A mice compared with 46.2±13.1 ng.mL in SOD1 mice (Mann-Whitney test, p = 0.035). As disease progressed, plasma NfH^SMI34^ levels continued to be lower in arimoclomol treated than vehicle treated SOD1 mice. Thus, the mean ± S.E.M NfH^SMI34^ levels in SOD1+A mice compared with SOD1 mice were 66.3±23.7: 81.8±15.8 (SOD1+A: SOD1) ng/mL at 90 days; 124.8±18.8: 155.4±19.3 ng/mL at 105 days and 231.7±23.9: 328.4±40.3 ng/mL at 120 days. However, the reduction in NfH^SMI34^ level in symptomatic SOD1+A mice compared with SOD1 mice only reached significance at 120 days (90 days p = 0.154, Mann-Whitney test; 105 days, p = 0.264, *t*-test; 120 days, p = 0.048, *t*-test; Fig. 6).

Similarly, plasma levels of NfH^SMI35^ were lower in SOD1+A mice compared with SOD1 mice at all stages of the disease (Fig. 6B). In contrast to NfH^SMI34^ levels, the assay for variably-phosphorylated NfH did not detect any significant effects of arimoclomol at pre-symptomatic stages. However, levels of NfH^SMI35^ in SOD1+A mice at 65 days of age were more consistent between different animals and at a lower level than observed in SOD1 mice, where there was a greater variation in plasma NfH^SMI35^ levels. Thus, the mean±S.E.M levels of plasma NfH^SMI35^ in SOD1+A and SOD1 mice (SODA: SOD) were 35.1±10.3: 74.0±20.8 ng/mL at 65 days; 55.4±15.5: 68.8±14.8 ng/mL at 90 days; 77.0±10.1: 104.8±17.0 ng/mL at 105 days and 92.0±15.5: 161.0±21.5 ng/mL at 120 days (Mann-Whitney test, p-values: 0.391; 0.327; <0.001 and 0.009 at 65, 90, 105 and 120 days, respectively; Mann-Whitney test).

Furthermore, a comparison of the relative plasma levels of the two NfH phosphoforms revealed that NfH^SMI34^ levels were significantly lower in SOD1+A mice than SOD1 mice at 65 days of age (Mann-Whitney test, p = 0.015). Furthermore, in SOD1+A mice, levels of NfH^SMI34^ became greater than NfH^SMI35^ in general in later stages, compared with SOD1 mice, and became significant at 105 days (p = 0.04). Thus, the disease modifying effects of arimoclomol in SOD1 mice were reflected in lower levels of both NfH phosphoforms as well as a more even composition of plasma NfH phosphoforms than in vehicle treated SOD1 mice.

### The Reduction in Plasma NfH Levels Reflect the Improvement in Muscle Force, Motor Unit and Motor Neuron Survival in Arimoclomol Treated SOD1 Mice

In order to determine if changes in plasma NfH levels could be used to detect the beneficial effects of arimoclomol, we first established that arimoclomol did in fact improve disease progression in SOD1+A mice, as we have previously reported [29], [30]. The results are shown in Table 1, which shows the mean muscle force, motor unit survival and motor neuron survival in SOD1+A mice and vehicle treated SOD1 littermates, at 105 and 120 days of age. The results confirm that there is a significant improvement in force, functional motor unit survival and the number of motor neurons that survive in the sciatic motor pool in SOD1+A mice compared with their vehicle treated SOD1 littermates, at both at a symptomatic (105d) but more clearly at a late stage (120d) of disease. These results also show that arimoclomol largely prevents the decline in muscle function and motor neuron survival that occurs in untreated SOD1 mice between 105 and 120 days of age.

**Table 1:** Arimoclomol delays disease progression in SOD1 mice.

Age (days)	Group	Maximum TA Tetanic Force(g ± sem) (n = muscle examined)	EDL MU survival (n ± sem)(n = muscle examined)	MN survival (%WT) (n = sciatic motor pool examined)
105	SOD1 SOD1+A	21.1±3.0 (16) 49.2±9.9 (11)**	12.6±0.5 (15) 14.8±0.5 (12)**	44.8 (10) 58.0 (10)**
120	SOD1 SOD1+A	19.5±3.5 (12) 36.9±9.0 (10)**	10.7±0.7 (11) 14.0±0.6 (10)**	29 (10) 65 (10)**

The Table shows the maximum TA tetanic force, EDL Motor Unit survival and Motor Neuron Survival in vehicle treated SOD1 and arimoclomol treated SOD1+A mice at 105 and 120 days of age. The results are the mean ± sem. **p<0.01.

## Discussion

In this study, we have validated plasma NfH levels as a marker of late stage disease progression and treatment response in an animal model of ALS. Plasma is a readily available biological fluid that is easy to sample in longitudinal studies of progressive neurodegenerative disorders such as ALS. As shown in Fig 1, our results show that plasma NfH levels increase significantly during overall disease progression between 65–120 days in the SOD1^G93A^ mouse model of ALS (Friedman test: p<0.0001) and that this increase in plasma NfH correlates with the loss of muscle force, decline in motor unit survival and most significantly, with the extent of motor neuron degeneration that occurs during later stages of disease. Moreover, our results also show that plasma NfH levels are a sensitive readout of late stage disease severity that can reflect the effects of a disease modifying therapy. Thus, in SOD1 mice treated with arimoclomol, we not only observed a significant delay in disease progression as previously reported [29], [30], reflected in an improvement in muscle force, motor unit and motor neuron survival, but we also detected a reduction in plasma NfH levels compared with vehicle treated age-matched SOD1 littermates. Arimolcomol is currently in a Phase II/III clinical trial in ALS [31], [33], [34]. The findings of this study therefore may represent an important step in the development of an easily accessible, comprehensive marker for ALS that may have utility in future clinical trials, particularly since patients are usually diagnosed and recruited into trials well after the onset of symptoms.

Although previous reports have suggested that NfH levels may be a candidate biomarker for ALS and other progressive neurodegenerative diseases including AD and PD [21], [22], [27], [35], most of these studies have examined CSF as the target biological fluid. This approach necessitates the use of a relatively invasive procedure i.e. lumbar puncture, which is not suitable for repeated sampling in longitudinal assessment of disease progression in individuals suffering from ALS. Analysis of plasma NfH is the obvious solution to this problem, but this can be technically challenging due to the so-called “hook effect”. This is caused by the tendency of neurofilaments to aggregate, resulting in masking of its epitopes and consequently, lower and un-representative yields in commonly used ELISA techniques [28]. The “hook effect” has to date, therefore prevented the reliable measurement of neurofilament levels in ALS and other diseases where NfH aggregate formation occurs by standard immunoassay. In this respect, the methodology used in this study to determine plasma NfH levels is unique, in so far it is the first specifically devised to overcome the ‘hook effect’ and, unlike previous methods, is sensitive enough to even detect NfH in plasma of WT mice [28].

Although the anatomically defined blood-brain barrier (BBB) and the functionally defined blood-CSF barrier (BCB) limit CNS-to-blood transfer, molecules expressed in the CNS can still enter the blood under both normal and pathological conditions. There is growing evidence for impaired BBB/BCB integrity in both SOD1 mice and post-mortem spinal cord tissue of ALS patients [36]. It is therefore not surprising that neurofilaments leak into the circulation in both SOD1 mice and ALS patients. The method we have recently developed [28] and used in this study was sensitive enough to detect both hyperphosphorylated (NfH^SMI34^) and variably-phosphorylated (NfH^SMI35^) forms of NfH in plasma of WT animals at all ages studied. Although the two forms of NfH were present in approximately equal amounts, the detection of hyperphosphorylated NfH in the plasma of healthy WT mice shows that the presence of hyperphosphorylated NfH in the peripheral blood is not in itself indicative of pathology. Moreover, since NfH has been detected in several pathological conditions [9], *its presence is not disease specific and it cannot therefore be used as a primary diagnostic biomarker for neurodegenerative conditions such as ALS*. However, plasma NfH levels do not reflect the earlier stages of disease in SOD1 mice, although we did observe a far larger variation in plasma NfH levels than in WT mice, even prior to symptom onset (65 days), when the NfH levels were not statistically different in SOD1 and WT mice (Fig. 1). Plasma NfH levels increased significantly in SOD1 mice between 105 to 120 days of age (Fig. 1). The sensitivity and reliability of the method used in the present study is highlighted by a comparison of our results with those of previous reports investigating plasma NfH as a potential biomarker of ALS. Thus, one study failed to detect any NfHs in plasma from WT mice and reported NfH levels in end-stage SOD1^G93A^ mice on average below 75 ng/mL [37] and the other study only detected NfH in plasma of end-stage SOD1^G93A^ mice at very low levels (on average, 8 ng/mL) [38]. In contrast, using the method employed in the present study, plasma NfH levels can be detected in WT mice at all ages studied, as well as in presymptomatic and end-stage SOD1^G93A^ mice, in which mean NfH levels rise to as high as 318.11 ng/mL [28].

In addition to examining changes in plasma NfH levels in SOD1^G93A^ mice, we also compared the relative levels of the two different NfH phosphoforms, NfH^SMI35^ and NfH^SMI34^. Our results show that at 65 days of age, in both WT and SOD1 mice, the plasma levels of NfH^SMI35^ and NfH^SMI34^ were similar to each other. However, by 90 days, a clear change in the relative levels of the two NfH phosphoforms was detected, with an increase in NfH^SMI34^ levels and a decline in NfH^SMI35^. It is possible that this change in the relative level of hyperphosphorylated NfH is a better indicator of pathology than simply the presence of this phosphoform *per se*. Thus, our results i) demonstrate the early involvement of NfH in pathology in SOD1 mice, ii) reveal the true abundance of NfH in the plasma of these mice and iii) suggest that an increase in the ratio of hyperphosphorylated NfH: variably phosphorylated NfH is indicative of pathology, at least in the SOD1^G93A^ mouse model of ALS.

In order to examine whether the increase in plasma NfH was a good reflection of the decline in neuromuscular function that occurs during disease progression in SOD1 mice, we performed a correlation analysis of plasma NfH levels with longitudinal and acute outcome measures. We found a strong-moderate inverse correlation between plasma NfH levels (both NfH^SMI34^ and NfH^SMI35^) and grip strength in SOD1 mice between 65–120 days of age. Grip strength is a general functional test widely used to evaluate motor function in mouse models [39]–[41]. However, grip strength is an overall measure of neuromuscular function that predominantly reflects the function of forelimb muscles, which are affected relatively late in disease progression in SOD1 mice. We therefore also correlated NfH levels with quantitative, reproducible physiological assessments of neuromuscular function in hindlimbs. We observed a moderate-strong correlation in the decline in muscle force (Fig. 3D) and increased plasma NfH and a mild correlation between plasma NfH and EDL motor unit loss (Fig. 4C) in SOD1 mice. The mild correlation between NfH levels and motor unit survival is likely to be a reflection of the relatively late involvement of the EDL muscle in disease in SOD1 mice [30], [32], so that motor unit loss in other muscles such as TA which is affected earlier and to a greater extent than EDL, may correlate better with NfH levels than EDL, but are technically more difficult to accurately estimate. Thus, the extent of motor unit loss in any individual muscle may not be a good measure of overall disease progression. Among the most direct measures of disease progression in ALS is the extent of motor neuron survival. It has long been thought that the loss of motor neurons in ALS is linear [42] and in the present study, morphological assessment of motor neuron survival at different ages reveals that motor neuron survival declines linearly in SOD1 mice. More importantly, we found a strong correlation between the extent of motor neuron degeneration and plasma NfH levels in SOD1 mice (Fig. 5C). These results show that plasma NfH levels, at least detected by the method described in this study, can be used to reflect the extent of motor neuron degeneration. Thus, our data clearly shows that longitudinal measurement of plasma NfH levels in SOD1 mice reveals that plasma NfH levels increase as disease progresses, particularly in later stages between 105–120 days which is also the most critical period of disease progress in transgenic SOD1^G93A^ mice model, and this increase in NfH levels correlate with the decline in muscle force and the death of motor neurons.

However, it should be noted that, there is likely to be a significant delay between the time that the first physical manifestations of disease occur (such as muscle denervation [43]), and when these result in functional deficits (such as reduced muscle force), and the appearance of Nfs in the peripheral blood. Thus, when the timeline between muscle denervation, axonal degeneration, formation of NfH-containing aggregates, MN death and the eventual entry of NfH into peripheral blood is taken into account, it is not surprising that there is a discrepancy in the time between the detection of early physical manifestations of the disease (90 days) and the first significant elevation in plasma NfH levels (105 days). However, the invasive physiological and morphological approaches used to determine the extent of disease in SOD1 mice in this study, although very sensitive, quantitative and accurate, are irreversible and not practical for longitudinal follow-up in mice. Moreover, these measures might not be clinically relevant for human as they may not reflect the state of disease in patients when they first present to their physician with symptoms. Therefore, the correlations we report between the increases in plasma NfH levels and the decline in neuromuscular function may provide a safe and easy measure for the evaluation of ***later*** disease progression in ALS.

Furthermore, our results also show that plasma NfH levels reflect the disease modifying effects of arimoclomol in SOD1 mice. In confirmation of our previous findings 29,30, we found that treatment with arimoclomol significantly delays the decline in neuromuscular function and death of motor neurons in SOD1 mice (Table 1). Furthermore, plasma NfH levels were in general lower in arimoclomol treated SOD1+A mice than vehicle treated SOD1 littermates at all ages studied, but most significantly at later stages of disease, from 105–120 days of age (Fig. 6). Hence, at 65 days, a pre-symptomatic age, plasma levels of NfH^SMI34^ were significantly lower in arimoclomol-treated SOD1+A mice compared with vehicle treated SOD1 littermates, possibly due to the early beneficial effects of arimoclomol in the periphery, at the neuromuscular junction (NMJ). Arimoclomol acts as a co-inducer of the heat shock response, so that it only acts in cells under conditions of cellular stress to augment the heat shock response (HSR; [44]). Thus, although the neuroprotective effects of arimoclomol in SOD1 mice only manifest later in the disease when motor neurons are under considerable stress [29], [30]), our recent results indicate that its beneficial effects in the periphery manifest earlier in the disease, prior to symptom onset. It has now been established that the earliest physical manifestation of disease in SOD1 mice occurs at the NMJ, where significant muscle denervation occurs prior to any motor neuron death [43]. Our recent work has shown that this early denervation is accompanied by an increase in the HSR and correspondingly, in arimoclomol treated SOD1 mice, this stress response is augmented, resulting in a delay in muscle denervation [45]. Therefore, since the levels of NfH remain steady in WT mice throughout the study, the difference in plasma NfH levels observed in SOD1+A and SOD1 mice at 65 days of age is likely to be the result of Arimoclomol’s early beneficial effects in the periphery and the maintenance of neuromuscular contacts.

However, as shown in Fig. 6 and Table 1, the significant improvement in muscle force, motor unit and motor neuron survival observed from 105 days of age in arimoclomol treated SOD1+A mice compared with their vehicle treated SOD1 littermates is reflected in a decrease in plasma NfH levels. Although the significance of reduction of NfH^SMI35^ observed from 105 days was in line with the physiological improvement, the reduction of NfH^SMI34^ became significantly reduced only at 120 days of age. However, further analysis of the levels of the two NfH phosphoforms showed that the relative increase in the more pathological plasma NfH^SMI34^ compared with NfH^SMI35^ occurred significantly later in SOD1+A mice than SOD1 mice (data not shown). It is likely that the difference in the timing of the effects of arimoclomol treatment on NfH^SMI35^ and NfH^SMI34^ levels may be related to their different levels of phosphorylation. Since the stability of NfH increases with the degree of phosphorylation [10], it will take longer for proteases to cleave hyperphosphorylated NfH compared with variable-phosphorylated NfH. Because neurofilament aggregates containing a large quantity of hyperphosphorylated NfH are a hallmark of disease in SOD1 mice, it will take longer for hyperphosphorylated NfH levels to decrease following treatment with arimoclomol compared with variable-phosphorylated NfH.

Taken together our results show that the late stage decline in neuromuscular function and motor neuron survival in SOD1 mice is correlated with an increase in plasma NfH phosphoform levels, and furthermore, that plasma NfH phosphoform levels reflect the improvement in disease phenotype induced by treatment with arimoclomol. Plasma NfH phosphoform levels may therefore be a useful marker to determine late stage disease progression in ALS and that may eventually be used as a sensitive outcome measure in clinical trials, particularly since ALS patients are likely to exhibit significant disease symptoms by the time of enrolment. The current outcome measures used in clinical trials are not particularly sensitive to change over disease progression and usually require a long period of observation, typically longer than 1 year. Even measurements that are capable of detecting the rapid degeneration of motor neurons in ALS, such as MUNE [46], have drawbacks, such as the need for specialist training, equipment and complex statistical analysis, together with the lack of a gold standard method, which prevents the widespread use of MUNE as an outcome measure in ALS [47]. Moreover, as the results of this study show, the selection of specific individual muscles for MUNE may not always give an accurate reflection of the extent of disease progression in other muscle groups variably affected by disease.

Accurate and sensitive assessment of plasma NfH phosphoform levels may therefore provide a quick and easy readout of disease progression in individual patient in future clinical trials. In addition, plasma NfH levels may also serve as a safety biomarker as a rapid increase in plasma NfH phosphoform levels following administration of test drugs might indicate a detrimental treatment effect [48]. The use of plasma NfH as an outcome measure may therefore help to improve the safety of clinical trials by speeding up the time taken to detect deleterious effects and by reducing the time taken to complete these studies, hence reducing costs of Phase III trials which account for 70% of costs of clinical drug development in ALS [49]. Whether our findings in an animal model of ALS can be translated to ALS patients will become clear through further longitudinal investigation on cohorts of ALS patients. If the results observed in this study in an animal model of ALS are translated into patient samples, accurately measured plasma NfH phosphoform levels may become a valuable biomarker of disease progression, especially in later stages, for the ALS community.

## Materials and Methods

### Experimental Animals

All animals were bred and maintained by Biological Services in the UCL Institute of Neurology. The experiments described in this study were carried out under licence from the UK Home Office and following approval from the UCL Institute of Neurology’s Ethical Review Panel. Transgenic mice expressing human SOD1^G93A^ mutant protein (TgN[SOD1-G93A]1Gur; Jackson Laboratories, Bar Harbour) were maintained by breeding male heterozygous carriers with female (C57BL/6× SJL) F1 hybrids. The presence of the SOD1^G93A^ mutation was confirmed by PCR reaction from ear biopsies in all mice at the age of 3 weeks. In order to obtain maximum blood sample volume of mice between 65–120 days of age whilst still complying UK Home Office Regulations (Less than 15% of the blood volume should be removed in any 30-day period, i.e. 10.5 ml/kg) [50], only male mice were examined in this project.

### Experimental Groups. Depending on Genotype, the Mice were Randomly Assigned to One of 3 Groups

1. Transgenic mice carrying human SOD1^G93A^ mutant protein treated daily with arimoclomol (10 mg/Kg; i.p) from 35 days of age (SOD1+A mice).
2. Transgenic mice carrying human SOD1^G93A^ mutant protein treated daily with vehicle (sterile saline; i.p) from 35 days of age (SOD1 mice).
3. Untreated wild type, age-matched littermates (WT mice).
4. Arimoclomol (kind gift of Biorex R&D Co., Hungary) was dissolved in sterile saline (2 mg/ml) and the solution stored at 4°C for a maximum of 1 week.

#### Longitudinal biomarker study

In the longitudinal study of plasma NfH levels, arimoclomol treated SOD1 mice (SOD1+A; n = 19), vehicle treated SOD1 mice (SOD1; n = 19) and untreated wild-type (WT; n = 13) mice were repeatedly examined at various stages of disease progression ranging from early symptomatic to end-stage disease.

#### Plasma collection

Blood sampling was carried out in mice from each experimental group at 65, 90, 105 and120 days of age, representing pre-symptomatic, early-symptomatic, late-symptomatic and late stage disease. At 65, 90 and 105 days, the mice were placed in a recovery chamber (Peco Service; V1200), set at 38.5°C, for 15 minutes and then transferred into a plastic tube with the tail exposed. Blood was collected from the tail vein into an EDTA-coated tube (BD Microtainer®, K2E). At 120 days, blood was collected by a cardiac puncture, under terminal anaesthesia using pentobarbital. Each tube was then centrifuged at 14,000 rpm for 8 min. The plasma was collected and protease inhibitor added (Sigma; v/v: 1/100). Each sample was then aliquoted and stored at −80°C until further analysis. In total, plasma samples collected from 19 SOD1 mice, 19 SOD1+A mice, and 13 WT mice at each time point were used for further analysis in this study.

### Functional Assessments

#### Grip strength

In the same mice selected for plasma collection, grip strength was determined prior to blood collection. According to the manufacturer’s instruction (Bioseb, BIO-GS3), mice were placed on a horizontal grid and pulled by the tail against the direction of the force gauge until the animal released the grid. An average of four readings was obtained at each occasion.

#### Acute in vivo physiological assessment of neuromuscular function and motor unit survival

In a separate set of mice, functional analysis of hindlimb muscle function was undertaken at 105 days of age, corresponding to a late symptomatic stage of disease. WT (muscle n = 10), SOD1 (muscle n = 12) and SOD1+A (muscle n = 16) mice were deeply anesthetised (4.5% chloral hydrate; 1 ml/100 g of body weight, i.p.) and prepared for *in vivo* analysis of isometric muscle force (as described in Kalmar et al., 2008 [30]). The distal tendons of the Tibialis Anterior (TA) and Extensor Digitorum Longus (EDL) muscles in both hindlimbs were dissected free and attached by silk thread to isometric force transducers (Dynamometer UFI Devices, Welwyn Garden City, UK). The sciatic nerve was exposed and sectioned. The length of the muscles was adjusted for maximum twitch tension. The muscles and nerve were kept moist with saline throughout the recordings and all experiments were carried out at room temperature. Isometric contractions were elicited by stimulating the nerve to TA and EDL using square-wave pulses of 0.02 ms duration at supra-maximal intensity, via silver wire electrodes. Contractions were elicited by trains of stimuli at frequencies of 40, 80 and 100 Hz. The maximum tetanic tension was measured using a computer and appropriate software (PicoScope).

The number of motor units innervating the EDL muscles was also determined by stimulating the motor nerve with stimuli of increasing intensity, resulting in stepwise increments in twitch tension due to successive recruitment of motor axons with increasing stimulus thresholds. The number of stepwise increments was counted to give an estimate of the number of functional motor units (MUNE) present in each muscle.

#### Morphological assessment of motor neuron survival

Following physiological assessment of muscle function, the mice (n = 5 per group) were terminally anaesthetised and perfused transcardially with saline followed by fixative containing 4% paraformaldehyde (PFA). The spinal cords were then removed and postfixed in 4% PFA and cryopreserved in 30% sucrose overnight at 4°C. Transverse sections (20 µm) of the fixed lumbar spinal cords (L3–L6) were cut on a freezing cryostat and collected serially onto glass slides and subsequently stained for Nissl (gallocyanin). Large polygonal neurons, with a minimum diameter of 20 µm, within the sciatic motor pool, which had a clear nucleolus and a distinct Nissl-dense cytoplasm were counted in every 3^rd^ section, in order to prevent counting the same neuron twice in consecutive sections [51].

### NfH ELISA

We have recently developed a sensitive, reproducible method for the detection of NfHs in plasma, which breaks-up NfH aggregates, thereby allowing reliable quantification of plasma NfH levels using ELISA [28]. In this ELISA, NfH levels in plasma from mice in each experimental group were determined using mouse monoclonal anti-NfH antibodies, SMI-34R and SMI-35R (Covance, USA) as the capture antibodies, rabbit polyclonal anti-Neurofilament 200 (N4142; Sigma, UK) as the detector antibody, and horseradish peroxidase (HRP)-labelled swine polyclonal anti-rabbit antibody (P0217; DAKO, Denmark) as the reporter antibody. NfH detected with the SMI-34R capture antibody is labelled as NfH^SMI34^, while NfH detected with the SMI-35R capture antibody is labelled as NfH^SMI35^. For a full description of this 4-layer sandwich ELISA and the reagents used see [28].

#### Analytical procedure

The microtitre plates were coated with 100 µl of capture antibodies, either SMI-34R or SMI-35R, in 0.05 M carbonate buffer, pH 9.5 (w/v, 2/10000), at 4°C overnight. The plates were rinsed once in Barb_2_EDTA buffer (13.1 g Sodium Barbitone, 2.1 g Barbital, 0.25 g EDTA) containing 0.05% Tween 20 and 0.1% BSA (wash solution) and then blocked with 150 µl of Barb_2_EDTA buffer containing 1% BSA at room temperature (RT) for 1 hour. Meanwhile, 5 µL of original plasma was added to 35 µL of Barb2EDTA buffer containing 0.5 M urea and mixed well at RT for 1 hour. After 2 rinses with wash solution, 95 µl of Barb_2_EDTA buffer containing 0.1% BSA (sample diluent) was added into each well. Five microliters of standard was added to standard wells, and sample wells were loaded with 5 µL of the diluted urea-treated plasma. Quality controls were also included, which consisted of pre-prepared NfH samples of various concentrations. All samples, standards and quality controls were loaded in duplicate. Plates were then incubated for 1 hour (RT) on a shaker. After washing (3×5 mins), 100 µl of detector antibody (w/v. 10/10000 in sample diluent) was added into each well and incubated for 1 hour on a shaker (RT). Plates were then washed (3×5 mins) before 100 µl of reporter antibody (w/v, 10/10000 in sample diluent) was loaded into each well and incubated for 1 hour on a shaker (RT). After washing (6×5 mins), 100 µl of TMB substrate was added into each well and incubated for approximately 20 minutes in the dark on a shaker (RT). The reaction was then stopped by adding 50 µl of 1 M HCl into each well. The absorbance was read immediately at 450 nm, with 750 nm as the reference wavelength, on an Omega plate reader (Software version: 1.02; BMG LABTECH).

#### Data analysis

Measurements with a coefficient of variation (CV) value higher than the assay limit (10%; [52]) were repeated. Quality control samples were used throughout and absorbance readouts from different microtitre plates were adjusted to the quality control readout to allow comparison of results across different plates.

### Statistical Analysis

Statistical analysis was carried out using SPSS software (V17). The normality of data was checked, using a Kolmogorov-Smirnov test, to determine the approach of parametric or nonparametric analysis. The repeated Friedman test was used for analysis of NfH levels within groups in the longitudinal biomarker study. The Mann-Whitney test or *t*-test, according to the normality of data, was used for analysis of NfH levels between groups at each time point (WT v.s. SOD1; SOD1 v.s. SOD1+A), and for analysis of the levels of the two NfH phosphoforms in each group, at different time points (NfH^SMI34^ v.s. NfH^SMI35^). Bivariate correlation analysis, using Spearman’s correlation coefficient, was examined for plasma NfH levels and grip strength. A minor, moderate and strong correlation is considered if Spearman’s rho (R) is <0.3, 0.3–0.5,>0.5, respectively. Statistical significance was set at p<0.05. Exponential analysis was used to determine the correlation of plasma NfH levels and functional readouts in SOD1 mice at various disease stages. A strong correlation was considered when the coefficient of determination (R^2^) obtained from the exponential analysis was greater than 0.8.
